# Genome-wide identification and analysis of *LOX* genes in soybean cultivar “Zhonghuang 13”

**DOI:** 10.3389/fgene.2022.1020554

**Published:** 2022-10-07

**Authors:** Jing Zhang, Cheungchuk Ng, Yan Jiang, Xianxu Wang, Shaodong Wang, Sui Wang

**Affiliations:** ^1^ Key Laboratory of Soybean Biology of Chinese Education Ministry, Northeast Agricultural University, Harbin, China; ^2^ State Key Laboratory of Tree Genetics and Breeding, Northeast Forestry University, Harbin, China

**Keywords:** soybean, Zhonghuang 13, lipoxygenase, genome-wide analysis, gene expression

## Abstract

Lipoxygenases (LOXs; EC1.13.11.12) are a family of iron- or manganese-containing dioxygenases that catalyze the oxygenation of polyunsaturated fatty acids (PUFAs) and play important roles in plant growth, development, and stress response. In this study, a total of 36 *LOX* gene family members were identified and annotated in Zhonghuang 13, a soybean cultivar bred by Chinese scientists in 2001. Sanger sequencing of the *GmLOX1*-coding sequence and colorimetric assays for the GmLOX1 protein showed that Zhonghuang 13 possessed the *GmLOX1* gene. These *LOX* genes are divided into three subfamilies: 9-LOX, type Ⅰ 13-LOX and type II 13-LOX. In the 13-LOX group, the number of *GmLOX* members was the highest. These *GmLOX* genes are unevenly distributed on chromosomes 3, 7, 8, 10, 11, 12, 13, 15, 16, 19, and 20. Most of the 13-*LOX* genes exist in the form of gene clusters, indicating that these genes may originate from tandem duplications. The analysis of duplicated gene pairs showed that *GmLOX* genes underwent purifying selective pressure during evolution. The gene structures and conserved functional domains of these genes are quite similar. Compared to the orthologous gene pairs of *LOX* genes between wild soybean (*Glycine soja* W05) and Zhonghuang 13, the sequences of most gene pairs are relatively conserved. Many cis-elements are present in the promoter region and are involved in stress response, growth and development, hormone response and light response. The tissue-specific gene expression of *GmLOX* genes was evaluated. Represented by *GmLOX1*, *GmLOX2*, and *GmLOX3*, which were expressed at extremely high levels in seeds, they showed the characteristics of specific expression. This study provides detailed information on soybean lipoxygenase gene family members in Zhonghuang 13, which lays a foundation for further research.

## Introduction

Lipoxygenases (LOXs) are iron- or manganese-containing dioxygenases (Feussner and Wasternack, 2002). LOXs of animals, plants and prokaryotes contain Fe as the catalytic metal, whereas fungi express LOXs with Fe or Mn (Wennman et al., 2016). LOXs can oxidize polyunsaturated fatty acids containing one or more 1Z, 4Z-pentadiene units to hydroperoxides (Brash, 1999; Song et al., 2016). The resulting hydroperoxides are further metabolized into biologically active oxylipins, including jasmonic acid (JA) and green leaf volatiles (GLV) (Upadhyay et al., 2019). LOXs and their products accumulate transiently upon developmental or environmental stimuli (Viswanath et al., 2020). JA can be involved in defense responses against abiotic and biotic environmental stresses (Vogt et al., 2013). LOXs are also known to play a decisive role in the production of volatiles that influence the flavor and aroma of floral fruit, which are important to the agri-food industry (Upadhyay et al., 2020). For instance, hydroperoxides, LOX-catalyzed products in mature soybean seeds, are converted to volatile compounds, which are associated with an unpleasant beany taste (Wang et al., 2020). In addition, LOXs have been associated with some processes in a series of developmental stages, including the mobilization of storage lipids during germination and fruit ripening (Porta and Rocha-Sosa, 2002; Viswanath et al., 2020). LOXs contain two major domains, a lipoxygenase domain at the C-terminus and a polycystin-1, lipoxygenase, alpha-toxin (PLAT) domain at the N-terminus (Song et al., 2016). The plant lipoxygenase domain is involved in iron binding and contains approximately 38 amino acids with five conserved histidines (Steczko et al., 1992). The PLAT domain, which contains an eight-stranded antiparallel β-barrel, is found in a variety of membrane- or lipid-associated proteins (Bateman and Sandford, 1999). This domain can bind to procolipase, which mediates membrane associations (Tomchick et al., 2001). According to the substrate oxygenated at either carbon atom 9 (9-LOX) or carbon atom 13 (13-LOX) of the hydrocarbon backbone of the fatty acid (Feussner et al., 2001), plant LOXs can be classified into two types, 9-LOX and 13-LOX (Kolomiets et al., 2001). Furthermore, it can be divided into I-LOXs and II-LOXs according to the consistency of sequence structure and whether there is a chloroplast targeting peptide: I-LOXs have high consistency but do not contain the targeted peptide that II-LOXs contain (Brash, 1999). All LOXs belong to the same gene family, and they are widely distributed. To date, many *LOX* genes have been identified, such as those in *Arabidopsis thaliana* (Park and Polacco, 1989), *Citrullus lanatus* (Liu J et al., 2020), *Fagopyrum tataricum* (Ji et al., 2020), *Arachis duranensis* (Song et al., 2016), *Malus pumila* (Vogt et al., 2013), *Spirodela polyrhiza* (Upadhyay et al., 2020) and *Populus L* (Chen et al., 2015). Zhou et al. (2020) demonstrated that overexpression of *CsLOX1* in cucumber may impair the production of endogenous JA. The function of *CsLOX1* may involve plant growth and development, such as shoot growth, male fertility and fruit development. Vogt et al. (2013) found that *MdLOX1a* and *MdLOX5e* were candidate genes involved in the production of apple fruit aroma volatiles. Chen et al. (2015) found that the expression patterns of *PtLOX* genes in response to stress were different in poplar. This result reflected that these genes may be involved in related functions of specific types of stress.

Soybean (*Glycine max* [L.] Merr.), one of the most important leguminous plants, is widely cultivated all over the world (Wang et al., 2020). The utilization of soybeans relates to multiple fields of production and life. Soybean’s oil content is approximately 20%, protein is approximately 40%, and it is a crucial raw material for food processing, which provides energy and nutrition (Chaudhary et al., 2015). In addition, soybean meal is used in a variety of animal feed, such as that for fish and pigs (Dunsford et al., 1989; Tomas et al., 2005), because it is high in protein with a good amino acid balance. Furthermore, soybeans are also used in industrial and pharmaceutical applications as well as in the production of biodiesel (Chaudhary et al., 2015). Soybean production has a great impact on human life. Studies on the evolution of the soybean genome indicate that the soybean underwent two whole genome replication events and has an ancient tetraploid genome (Schmutz et al., 2010). The soybean genome size is approximately nine times that of *A. thaliana* (Haas et al., 2003). At present, genome information of several soybean varieties has been identified (Schmutz et al., 2010; Shen et al., 2018; Liu Y et al., 2020). Williams 82, a soybean cultivar domesticated in America, was the first soybean genome to be sequenced, and the emergence of this reference genome made it possible to explore functional genomics in soybean (Chan et al., 2012). As an undomesticated relative of cultivated soybean, the sequencing of the wild soybean (*G. soja* W05) genome was helpful to explore the diversity of lost genes in cultivated soybean and provided a novel gene source for soybean breeding and improvement (Li et al., 2014). For cultivated soybeans, there are wide genetic differences among different varieties. The soybean cultivar “Zhonghuang 13” possesses the widest planting range in China (Liu J et al., 2020). The genome of Zhonghuang 13 was sequenced, and the reference genome represents the characteristics of Asian soybean (Shen et al., 2018). Compared with other reported plant genomes, the genome assembly of Zhonghuang 13 is more complete and more continuous (Shen et al., 2019). The elucidation of genome sequencing in Zhonghuang 13 is of great significance for further exploration of the trait-related genes of Asian soybean. Multiple LOX isozymes distinct from seed isozymes have been found in many tissues of soybean (Park and Polacco, 1989). The expression patterns of LOXs are different during the different stages from seed germination to plant maturity and in different parts of the plant (Veldink et al., 1977). Considering the potential roles of *LOX* genes, it is necessary to study the genome-wide analysis of *LOX* genes in Zhonghuang 13.

In this study, we identified the members of the *LOX* gene family using hidden Markov model (HMM) searches against a reference soybean genome of a soybean cultivar, Zhonghuang 13. For genes that were missing annotations, such as *GmLOX1*, Sanger sequencing and colorimetric assays were utilized to detect their existence at the transcription level and translation level, respectively. A phylogenetic tree was constructed with four outgroup species. Next, we analyzed the gene structures, conserved motifs, chromosomal distributions, collinearity, and cis-regulatory elements. Subsequently, we explored the evolution of *LOX* genes by comparing the discrepancy in *LOX* genes between wild soybean (*G. soja* W05) and Zhonghuang 13. Furthermore, we evaluated the expression of *GmLOX* genes in different parts of soybean to explore their spatial expression. Our results provide information on the soybean *LOX* gene family and lay a foundation for further study on the specific function of *LOX* genes in soybeans.

## Materials and methods

### Plant materials and treatment

The Zhonghuang 13 seeds were planted in a round pot with a diameter of 15 cm in a greenhouse in May 2021. Soybean plants grew naturally in the soil under greenhouse conditions. When the plants were at the R6 stage (pod stage), the roots, stems, leaves, flowers, pods, and seeds of the soybean plants were taken as the research materials [flowers were obtained at the R2 stage (flowering period)], placed in liquid nitrogen and stored at −80°C. Each treatment consisted of three biological replicates.

### Identification of *LOX* family members in Zhonghuang 13

The genome information of Zhonghuang 13 (Gmax_ZH13_v2.0) was downloaded from the Genome Warehouse in BIG Data Center under accession number GWHAAEV00000000.1 (Shen et al., 2019). To compare *LOX* genes between different species, we also downloaded the genome sequences of *Arabidopsis thaliana* (TAIR10), *Populus trichocarpa* (v4.1), *Vitis vinifera* (v2.1) and *Oryza sativa* (v7.0) from the Phytozome database (https://phytozome.jgi.doe.gov/). The genome data of Williams 82 (version *G. max* Wm82.a4.v1) were also downloaded from the Phytozome database to compare *LOX* genes in different soybean varieties. In addition, to acquire all the possible *LOX* genes, the HMM profiles of the lipoxygenase domain (PF00305) and the PLAT domain (PF01477) were downloaded from the Pfam database (http://pfam.xfam.org/). The protein sequences of *LOX* genes were searched against the HMMER program (v 3.3.2) (Finn et al., 2011). If both of these domains exist, the gene was recognized as an *LOX* gene. To ensure the accuracy of gene identification, the sequences were submitted to the Search Pfam site (http://pfam.xfam.org/search) for domain visualization and manual inspection to delete the unqualified genes. The isoelectric point (pI) and molecular weight (MW) of soybean LOXs were calculated by the Compute pI/Mw tool in ExPASy (https://web.expasy.org/compute_pi/). Protein subcellular localization prediction was performed by the WoLF PSORT website (https://wolfpsort.hgc.jp/).

The *GmLOX1* gene is an important soybean lipoxygenase gene. Primers were designed for the full-length *GmLOX1*-encoding sequence. The RNA of Zhonghuang 13 seeds was extracted and reverse transcribed into cDNA as a template. A 2×Taq PCR MasterMix II kit (*TIANGEN*, KT211-02, Beijing, China) was used to amplify the target sequence, and the PCR product was examined by DNA agarose gel electrophoresis with a 5,000 bp DNA marker (*Trans*, BM111-01, Beijing, China). The PCR product that matched the expected length was sent to GENEWIZ for Sanger sequencing. The colorimetric assay method mentioned in previous studies (Wang et al., 2020) was used to identify the presence of GmLOX1 protein in Zhonghuang 13 seeds, with Williams 82 and Dongfudou-3 being the positive and negative controls, respectively. Dongfudou-3, bred by Northeast Agricultural University, is a soybean cultivar lacking the GmLOX1 protein.

### Multiple sequence alignment and phylogenetic analysis

To investigate the protein sequences, multiple sequence alignment of the LOX protein sequences identified in five species was performed using the MAFFT program (version 7.490) (Katoh et al., 2002). A phylogenetic tree was created using the maximum likelihood (ML) method with 1000 ultrafast bootstrap replications by the IQ-TREE program (version 2.2.0) (Nguyen et al., 2015). The classification of LOX protein sequences depends on the characteristics of 9-LOX and 13-LOX. The phylogenetic tree was visualized using the Evolview (He et al., 2016) online site (https://www.evolgenius.info/evolview/).

### Gene structure and conserved motif prediction

The intron and exon localization information of *GmLOX* genes was extracted from genome annotation files. The Multiple Expectation Maximization for Motif Elicitation (MEME) program (Timothy and Bailey, 1994) (https://meme-suite.org/meme/tools/meme) was employed to confirm conserved motifs with the following parameters: the site distribution site was set to zero or one occurrence per sequence (zoops), the number of motifs was set to 10, and the minimum and maximum widths were 6 and 50, respectively. The Gene Structure View (Advanced) program in TBTools software (Chen et al., 2020) was used to visualize gene structure and conserved motifs.

### Chromosomal location and duplication analysis

The chromosomal location information of *GmLOX* genes was obtained from the soybean genome database. TBtools software was used to visualize the location of genes on chromosomes. Duplication analysis among the *GmLOX* genes was conducted with the MCScanX (Wang et al., 2012) tools in TBtools software. The parameters of BLAST (Camacho et al., 2009) were as follows: the *E*-value was set to 1e-5, the number of hits was set to 5, and the number of alignments was set to 5. When running the MCScanX program, the minimum block size was set to 5. KaKs_Calculator 3.0 (Zhang, 2022) was used to calculate the *K*_*a*_/*K*_*s*_ values of homologous gene pairs.

### Prediction of cis-regulatory elements in the *GmLOX* promoters

The 2,000 bp upstream sequences of the *GmLOX* gene coding sequences were obtained from genomic data of soybean and then uploaded to the PlantCARE database (http://bioinformatics.psb.ugent.be/webtools/plantcare/html/) (Lescot et al., 2002) with default parameters to predict stress- and hormone-responsive cis-regulatory elements. The visualization of the regulatory elements was performed with the Evolview online tool.

### Evolution of *LOX* genes between wild soybean (*G. soja* W05) and Zhonghuang 13

To analyze the evolution of *LOX* genes in wild soybean and cultivated soybean, genomic data of wild soybean (*G. soja* W05) were downloaded from DDBJ/ENA/GenBank under accession QZWG00000000 (Xie et al., 2019), and members of the *LOX* gene family of wild soybean were identified according to the above procedure (Song et al., 2016). Data from the identified duplicated blocks and orthologous gene pairs in two soybean varieties were obtained by the MCscanX tool in TBtools software. The One Step MCScanX function was used to process two genomic datasets, with the *E*-value set to 1e-5 and the number of BlastHits set to 5. In addition, the orthologous gene pairs of *LOX* genes were checked by BLAST. KaKs_Calculator 3.0 (Zhang, 2022) was used to calculate the *K*_*a*_ */K*_*s*_ values of coding sequences and the *K*_*n*_/*K*_*s*_ values of intro sequences and promoter sequences of the orthologous gene pairs of *LOX* genes. The MA method (Zhang B et al., 2006) was used for estimating *K*_*a*_/*K*_*s*_, and 0.618 was used as the neutral mutation rate for estimating *K*_*n*_/*K*_*s*_.

### Expression analysis based on RNA-Seq data in different tissues

The samples were sent to Wuhan Frasergen Bioinformatics Co., Ltd. on dry ice for library preparation and sequencing using the BGISEQ-500 platform. After data quality control by FastQC (v 0.11.9), raw reads were cleaned by fastp (v 0.23.1) (Chen et al., 2018) and 10 bases with poor quality at the starting position of reads, bases with base quality values less than 20, reads with lengths less than 90 bases, and reads that more than 20% did not meet the quality control standards were removed. The clean reads were aligned to the genome using HISAT2 (v 2.2.1) (Kim et al., 2015). SAMtools (v. 1.12) (Danecek et al., 2021) was used to convert SAM files to BAM files. The Trimmed Mean of M Value (TMM) normalization method was used to calculate transcripts per million (TPM) values. The TPM value after log10 processing was used to reflect the gene expression. The heatmap was drawn with Omicshare online cloud tools.

### RNA isolation and quantitative real-time PCR (qRT‒PCR)

The samples were pulverized in liquid nitrogen, and total RNA was extracted using a Generic Plant Total RNA Rapid Extraction kit (*Bio Teke*, RP3302, Wuxi, China) according to the manufacturer’s instructions. The concentration and purity of RNA were measured by an ultramicro nucleic acid analyzer (Nano400A). Genomic DNA was removed, and RNA (1 μg) was reverse transcribed into cDNA using the PrimeScript RT reagent Kit with gDNA Eraser (*Takara*, RR047A, Beijing, China). The Primer-BLAST tools in NCBI (https://www.ncbi.nlm.nih.gov/tools/primer-blast/) and the qPrimerDB online tools (https://biodb.swu.edu.cn/qprimerdb/) were used to select specific primers for 12 *GmLOX* genes and three internal reference genes. Detailed primer sequences are shown in Supplementary Table S1. Three technical replicate qRT-PCRs were performed with SYBR Green Realtime PCR Master Mix (*Toyobo*, QPK-201, Shanghai, China) in an AriaMx real-time PCR system G8830A for each biological replicate. The amplification conditions were as follows: pre-denaturation reaction at 95°C for 1 min, denaturation reaction at 95°C for 15 s, 45 cycles, extension reaction at 60°C for 1 min, with Rox as the reference dye. After the last polymerase chain reaction cycle, the melting curve was drawn under the conditions of 95°C for 15 s, 65°C for 1 min and 95°C for 15 s. The 2^−ΔΔCt^ method (Livak and Schmittgen, 2001) was used to calculate the gene expression data. According to the previous literature (Hu et al., 2009; Gao et al., 2017), we screened three internal reference genes, β-actin (*ACT*), cyclophilin (*CYP2*) and elongation factor 1-beta (*ELF1B*), which are involved in different biological regulatory mechanisms. After selecting the stably expressed internal reference genes, the geometric mean expression levels of the selected reference genes were used to normalize the expression levels of our target genes.

## Results

### Identification of LOX family members in Zhonghuang 13

A total of 36 soybean LOX proteins were ultimately identified. All of the deduced proteins contained the lipoxygenase domain (PF00305) and the PLAT domain (PF01477) based on Pfam. According to the 10 *LOX* genes registered on the National Center for Biotechnology Information (NCBI) in soybeans, we found the same genes in Zhonghuang 13 through BLAST (Camacho et al., 2009). Other genes were named *GmLOX11*–*GmLOX36* according to their locations on chromosomes. Detailed information on the *GmLOX* genes identified in Zhonghuang 13 (including chromosome position, CDS length, protein length, MW, pI, and subcellular localization) is displayed in Table 1. The GmLOX protein lengths ranged from 807 (*GmLOX20*) to 928 (*GmLOX16*) amino acids. The predicted molecular weight of the GmLOX proteins varied from 90.58 (*GmLOX26*) to 104.82 (*GmLOX30*) kDa, and the pI values ranged from 5.62 (*GmLOX29*) to 8.98 (*GmLOX18*). Most of the GmLOX proteins were predicted to be localized within the cytoplasm and chloroplast, and several were located within the nucleus and vacuole.

**TABLE 1:** The sequence characteristics of *GmLOX* in Zhonghuang 13.

Gene name	Sequence ID	Chromosome position	CDS (bp)	Protein properties			
				Length (aa)	MW (KDa)	pI	Subcellular localization
*GmLOX1*	*SoyZH13_13G319810*	Chr13:44982461–44986290 (+)	2520	840	94.41	5.91	Cytoplasm
*GmLOX2*	*SoyZH13_13G319800*	Chr13:44974893–44979099 (+)	2601	867	97.27	6.21	Cytoplasm
*GmLOX3*	*SoyZH13_15G025600*	Chr15:2205906–2210381 (+)	2574	858	96.77	6.12	Cytoplasm
*GmLOX4*	*SoyZH13_13G319900*	Chr13:44986659–44993832 (−)	2562	854	96.46	5.76	Cytoplasm
*GmLOX5*	*SoyZH13_15G025800*	Chr15:2224297–2229640 (+)	2562	854	96.64	5.78	Cytoplasm
*GmLOX6*	*SoyZH13_07G006000*	Chr07:498989–504617 (+)	2580	860	96.32	6.22	Cytoplasm
*GmLOX7*	*SoyZH13_13G320000*	Chr13:45010781–45023174 (+)	2571	857	96.33	6.23	Cytoplasm
*GmLOX8*	*SoyZH13_07G006100*	Chr07:505204–509990 (+)	2595	865	96.82	5.78	Cytoplasm
*GmLOX9*	*SoyZH13_07G033500*	Chr07:2805676–2811904 (−)	2598	866	96.35	6.54	Cytoplasm
*GmLOX10*	*SoyZH13_08G181100*	Chr08:15399791–15404534 (+)	2601	867	97.17	6.02	Cytoplasm
*GmLOX11*	*SoyZH13_03G083300*	Chr03:29014398–29022135 (+)	2601	867	99.50	8.98	Nucleus
*GmLOX12*	*SoyZH13_03G216800*	Chr03:45589395–45596199 (−)	2577	859	97.65	5.8	Vacuole
*GmLOX13*	*SoyZH13_03G242900*	Chr03:47564621–47569448 (+)	2706	902	102.13	7.9	Cytoplasm
*GmLOX14*	*SoyZH13_07G005900*	Chr07:486511–492502 (+)	2604	868	97.66	5.85	Cytoplasm
*GmLOX15*	*SoyZH13_07G033700*	Chr07:2824026–2829883 (−)	2607	869	97.90	6.07	Nucleus, Cytoplasm
*GmLOX16*	*SoyZH13_07G038600*	Chr07:3341283–3347832 (−)	2784	928	104.34	7.09	Chloroplast
*GmLOX17*	*SoyZH13_08G097100*	Chr08:7977300–7982728 (−)	2766	922	103.67	7.96	Chloroplast
*GmLOX18*	*SoyZH13_08G180600*	Chr08:15338088–15343759 (+)	2475	825	92.72	7.62	Cytoplasm, Chloroplast
*GmLOX19*	*SoyZH13_08G180700*	Chr08:15351171–15356438 (+)	2586	862	99.12	6.12	Cytoplasm
*GmLOX20*	*SoyZH13_08G180900*	Chr08:15358268–15366700 (+)	2421	807	90.58	7.61	Cytoplasm
*GmLOX21*	*SoyZH13_08G181000*	Chr08:15371650–15377445 (+)	2604	868	97.77	5.67	Cytoplasm
*GmLOX22*	*SoyZH13_08G181300*	Chr08:15413707–15419033 (+)	2607	869	97.14	6.93	Chloroplast, Cytoplasm
*GmLOX23*	*SoyZH13_10G139800*	Chr10:41448973–41455237 (−)	2598	866	98.16	5.62	Cytoplasm
*GmLOX24*	*SoyZH13_10G229800*	Chr10:50246602–50254961 (−)	2646	882	100.34	6.38	Cytoplasm
*GmLOX25*	*SoyZH13_11G126100*	Chr11:9869417–9878631 (−)	2721	907	103.81	6.91	Chloroplast
*GmLOX26*	*SoyZH13_11G126200*	Chr11:9889118–9901809 (−)	2706	902	102.63	5.76	Chloroplast
*GmLOX27*	*SoyZH13_12G050600*	Chr12:4011753–4019794 (−)	2745	915	104.42	7.21	Chloroplast
*GmLOX28*	*SoyZH13_13G019300*	Chr13:10539113–10545882 (+)	2757	919	104.30	6.34	Chloroplast
*GmLOX29*	*SoyZH13_13G060200*	Chr13:18753956–18764277 (−)	2736	912	104.82	6.93	Nucleus
*GmLOX30*	*SoyZH13_15G025700*	Chr15:2212777–2217044 (−)	2571	857	96.38	6.34	Cytoplasm
*GmLOX31*	*SoyZH13_16G007400*	Chr16:723124–730185 (−)	2769	923	104.01	6.91	Chloroplast
*GmLOX32*	*SoyZH13_16G075600*	Chr16:9077939–9088191 (−)	2601	867	99.75	8.74	Nucleus
*GmLOX33*	*SoyZH13_19G247200*	Chr19:52083994–52088822 (+)	2700	900	101.46	7.62	Chloroplast
*GmLOX34*	*SoyZH13_20G048300*	Chr20:12602780–12611402 (+)	2580	860	97.25	5.66	Cytoplasm
*GmLOX35*	*SoyZH13_20G048700*	Chr20:12753774–12760351 (+)	2583	861	97.88	5.74	Cytoplasm
*GmLOX36*	*SoyZH13_20G131200*	Chr20:42643020–42651181 (+)	2577	859	97.92	6.21	Cytoplasm

Note: Gene names, named according to sequence IDS; chromosome localization, obtained from the gene annotation file of Zhonghuang 13; CDS, base pair length of derived coding sequence; Length (aa), deduced length of the polypeptide; Mw, molecular weight of the polypeptide; PI, isoelectric point of the polypeptide; subcellular localization, predicted using the WoLF PSORT website.

At present, there are three strategies for gene sequence annotation: *de novo* prediction, homology-based prediction and transcription-based prediction. The accuracy of these prediction methods is limited. It is necessary to manually determine the accuracy of *GmLOX* genes. We compared the *LOX* gene family members identified in Zhonghuang 13 with those identified in previous studies in Williams 82 (Song et al., 2016) and listed homologous gene pairs in Supplementary Table S2. There were three unpaired genes, *SoyZH13_10G229800* (*GmLOX24*), *Glyma.07G196800* and *Glyma.20G054000*. *SoyZH13_10G229800* was retained because its domain and protein sequence structure met the requirements of LOX protein. A BLAST alignment (*E*-value < 1e-5) was performed on Williams 82 genome data using the coding sequence of *SoyZH13_10G229800* as the query sequence. The results showed that a part of the alignment was on chromosome 10 and the other part was on scaffold_524 (identity >99%). It could be inferred that scaffold_524 should be attached to chromosome 10, and there may be an unannotated *LOX* gene on chromosome 10 in Williams 82. The corresponding regions of *Glyma.07G196800* and *Glyma.20G054000* in Zhonghuang 13 were also found by BLAST alignment. *Glyma.07G196800* corresponded to *SoyZH13_07G182200*, which resulted in premature termination of protein translation due to a single base mutation. The corresponding region of *Glyma.20G054000* was frame-shifted due to single base insertion. As a result, both regions lack the necessary structure for the *LOX* gene (verification information in Supplementary Figure S1). Based on comparison with *LOX* gene family members in Williams 82 and the support of RNA-seq data, we modified the *LOX* gene annotation information in Zhonghuang 13 (corrected the gene annotation information of *GmLOX12*, *GmLOX2* and *GmLOX35* and added the new gene *GmLOX1*). It should be noted that although these corrections refer to multiple model plants and experimental results, they cannot be guaranteed to be complete and accurate and need to be verified by further experiments in the future. The modified gene annotation file result has been uploaded to http://www.wangsui.net.cn/resource/database/public/plant/Glycine_max/ ZH13/Lox_gene_family/Gmax_ZH13_v2.0_corrected.v1.0.gff3.gz. The extraction results of the *GmLOX* genes coding sequences (CDSs), GmLOXs protein sequences and 2,000 bp of the nucleotide sequences upstream of the *GmLOX* translation initiation codon were in Supplementary Data S1–S3, respectively.

### Modified *GmLOX1* gene, Sanger sequencing and protein identification of *GmLOX1*

**FIGURE 1:**
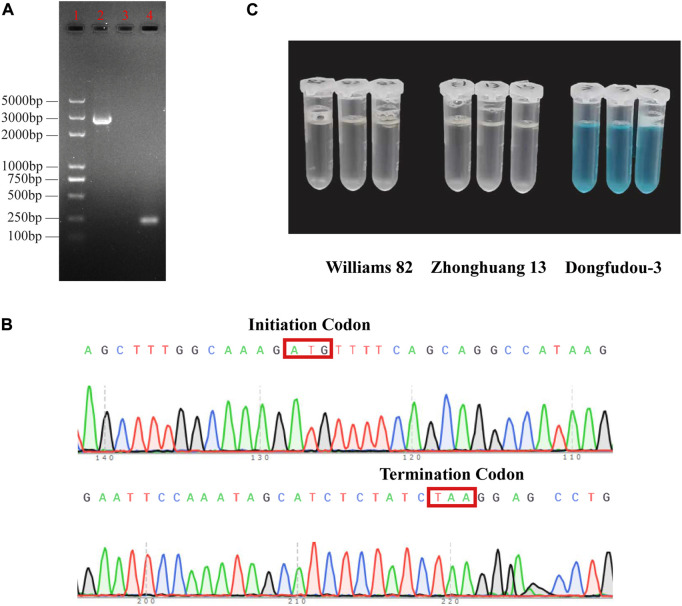
Identification results of the *GmLOX1* gene **(A)** Gel image showing PCR products of *GmLOX1* in Zhonghuang 13 seeds. **(B)** The initiation codon and termination codon in Sanger sequencing. **(C)** The detection results of the colorimetric assay method. Note: **(A)** 5,000 bp marker in lane 1, PCR products in lane 2, negative control in lane 3 and positive control in lane 4. The negative control used nuclease-free water as the cDNA template. The positive control used the primers for qRT‒PCR of *GmLOX1.* **(C)** The sample with GmLOX1 protein will be colourless; without GmLOX1 it will be blue.

**FIGURE 2:**
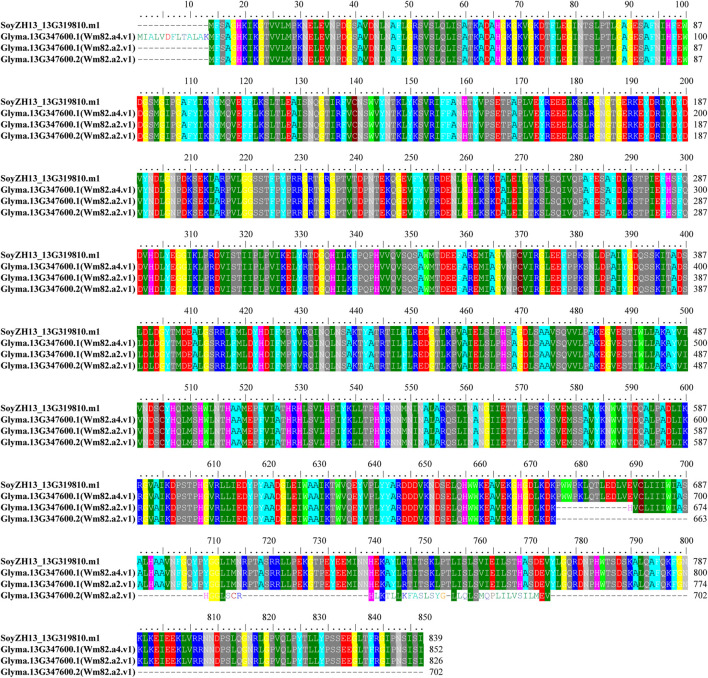
Alignment result of the modified GmLOX1 protein sequence in Zhonghuang 13 with different versions of GmLOX1 of Williams 82 Note: *SoyZH13_13G319810* is the sequence ID of *GmLOX1* in Zhonghuang 13. *Glyma.13G347600* is the sequence ID of *GmLOX1* in Williams82, and there is one transcript in Wm82.a4.v1 and two transcripts in Wm82.a2.v1.

To further confirm the information of *GmLOX1*, Sanger sequencing and colorimetric assays were used to detect its existence at the transcription level and translation level, respectively. The length of the *GmLOX1*-encoding sequence is 2,520 bp. The result of electrophoresis in agarose gel was consistent with the expected fragment length (Figure 1A). The raw sequencing results are provided in Supplementary Data S4. The full sequence of *GmLOX1* was sequenced. The initiation codon and the termination codon of sequencing are shown in Figure 1B **.** The results of the colorimetric assay showed that Zhonghuang 13 contained the GmLOX1 protein. As shown in Figure 1C, the negative control Dongfudou-3 is blue, and Zhonghuang 13 is colorless, the same as the positive control Williams 82. According to the sequencing results and the *GmLOX1* gene sequences in different versions of Williams 82, the annotation of the *GmLOX1* gene of Zhonghuang 13 was modified. The alignment result of GmLOX1 protein sequences between Zhonghuang 13 and different versions of Williams 82 is shown in Figure 2. The start position of *GmLOX1* in Zhonghuang 13 is the same as that in Williams 82.a2.v1, and the end position is the same as that in Williams 82.a4.v1. A single base discrepancy (Wm82.a4.v1 one more base than Wm82.a2.v1) caused the difference in GmLOX1 protein sequences between the two versions of Williams 82.

### Multiple sequence alignment and phylogenetic analysis

To acquire more information about the characteristics and obtain the number of members in each LOX subfamily, we compared the LOX proteins in five plant species (*A. thaliana*, *P. trichocarpa*, *V. vinifera*, *O. sativa* and *G. max*). We used the same method to identify LOX proteins in these species, and the numbers of *LOX* gene family members in *A. thaliana, P. trichocarpa*, *V. vinifera* and *O. sativa* were 6, 18, 11, and 11, respectively. Gene names corresponding to sequence IDs are shown in Supplementary Table S3. Soybeans have the highest number of members of the *LOX* gene family in the five species.

**FIGURE 3:**
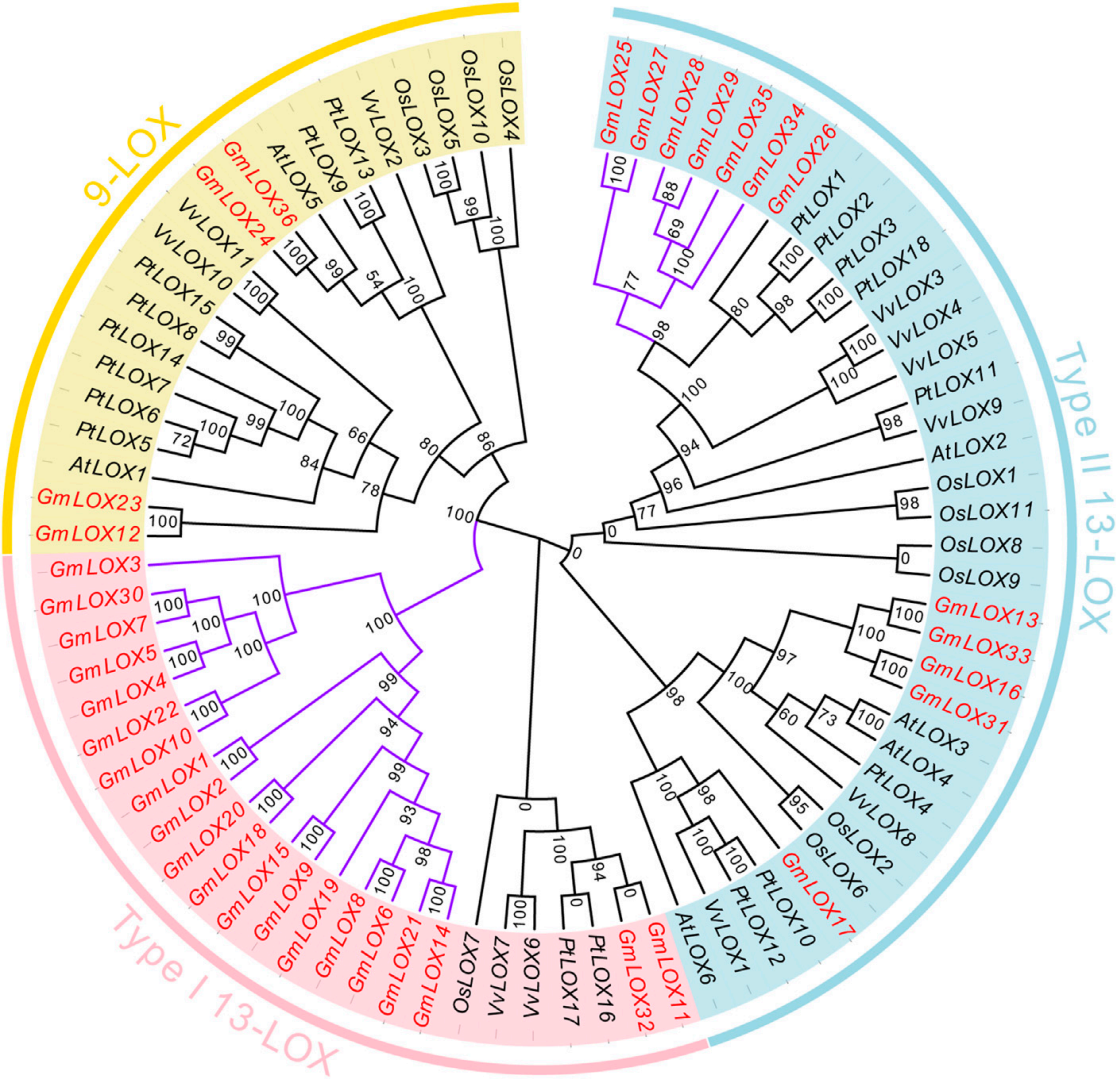
Phylogenetic analysis of GmLOX proteins in Zhonghuang 13 with other plant species Note: AtLOX: *Arabidopsis thaliana*. PtLOX: *Populus trichocarpa*. OsLOX: *Oryza sativa*. VvLOX: *Vitis vinifera*. Phylogenetic trees were constructed using the full-length amino acid sequences of LOX proteins from five species by the maximum likelihood (ML) method with 1000 ultrafast bootstrap replicates, and the ultrafast bootstrap support values are indicated on each node.

To clarify the genetic relationships of these *LOX* genes and which subfamily they belong to, a total of 82 LOX proteins in these five species were subjected to multiple sequence alignment**,** and an ML phylogenetic tree was constructed among 6AtLOXs, 11OsLOXs, 18PtLOXs, 11VvLOXs, and 36GmLOXs (Figure 3). According to the Bayesian Information Criterion (BIC), the Q.plant + I + G4 model was chosen as the best-fit model. The *LOX* genes that were reported in the paper (Umate, 2011; Chen et al., 2015; Song et al., 2016) have already been classified into three subfamilies: the type Ⅰ 13-LOX subfamily, type II 13-LOX subfamily, and 9-LOX subfamily. Here, based on the clusters displayed on the phylogenetic tree, we were able to classify GmLOX into the above three subfamilies. The number of members of GmLOX among the type Ⅰ 13-LOX subfamily, type II 13-LOX subfamily, and 9-LOX subfamily was 20, 12, and 4, respectively (the red font gene names are shown in Figure 3). We found that in the type Ⅰ 13-LOX subfamily, there are four times as many GmLOX members as the LOX members of other species combined, while there are no AtLOX members. The GmLOX members occupy an entire branch (the purple highlight branch shown in Figure 3). In the 9-LOX subfamily, the number of GmLOX members is relatively rare compared to that in other species. There is also a small branch that includes all GmLOX members in the type II 13-LOX.

### Gene structure and conserved motif prediction

**FIGURE 4:**
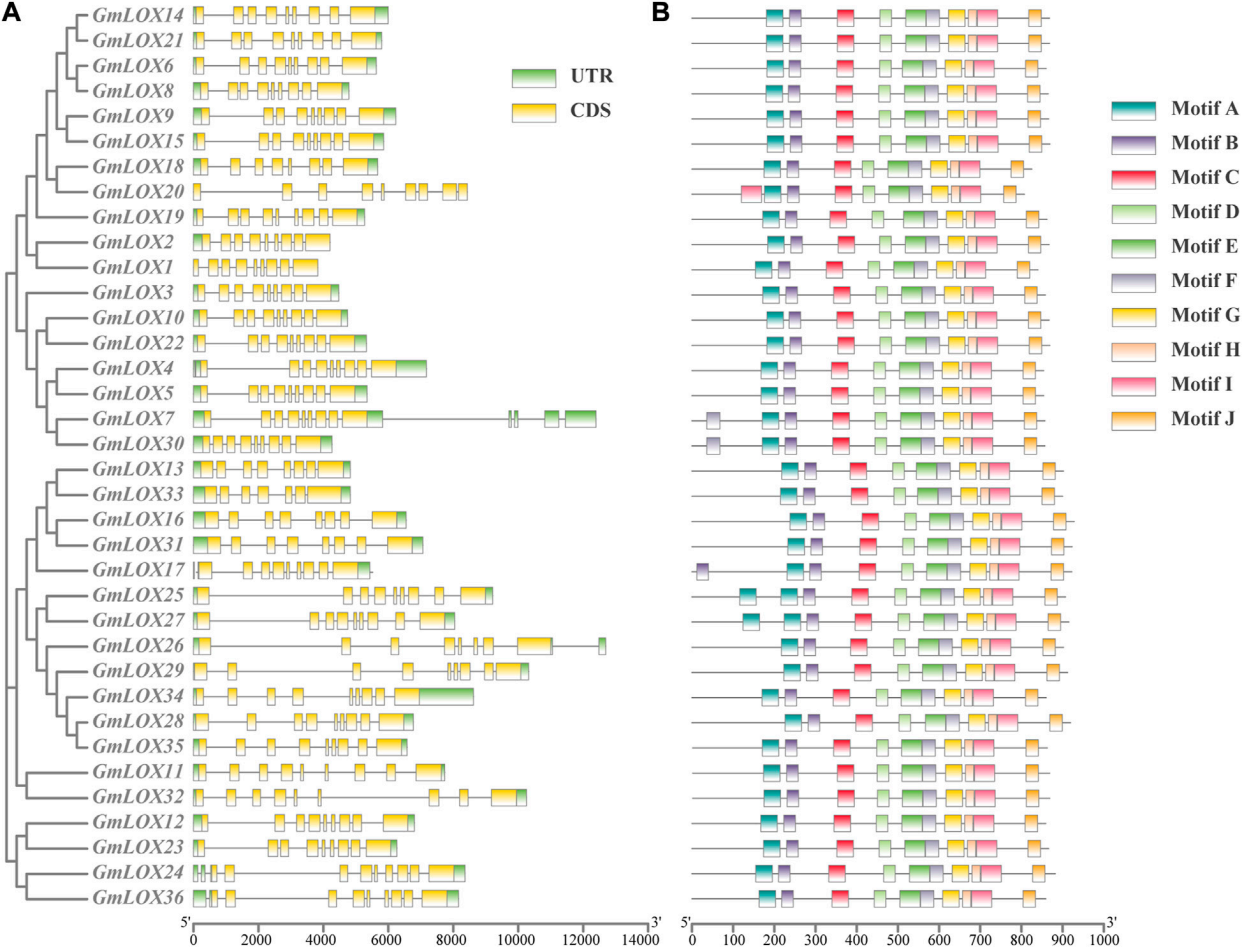
Gene structure analysis and conserved motif prediction of *GmLOX* genes in Zhonghuang 13 Note: **(A)** The result of gene structure analysis with a cluster analysis tree of polypeptide sequences by the ML method. CDS (coding sequence) regions are marked with yellow boxes, UTR (untranslated region) with green boxes and intron with black lines. **(B)** The motifs represented by different coloured boxes refer to the legend on the right. The scales at the bottom are used to measure the length of the sequence.

A separate phylogenetic tree was constructed using the 36 *GmLOX* coding sequences, and the intron and exon locations of each gene sequence were extracted and compared (Figure 4A) to acquire further insight into the gene structure of the soybean lipoxygenase gene family in detail. Each gene contains at least seven exons and at most 13 exons, with nine exons being the most common among the genes. The genetic structure of the soybean lipoxygenase gene was displayed similarly by comparing the position distribution of the *GmLOX* gene coding regions.

As shown in Figure 4B, 10 types of motifs were detected (Supplementary Figure S2). The motifs that were identified contained between 21 and 50 amino acids. All members shared 10 motifs, which indicates the high sequence similarity of GmLOXs, and all the motifs were arranged in the same order, which suggests that the motif locations of GmLOXs are conserved. For certain motifs, several genes possess more than one motif. GmLOX20 has an extra motif I, GmLOX17 has an extra motif B, GmLOX7 and GmLOX30 have an extra motif F, and GmLOX25 and GmLOX27 have an extra motif A.

### Chromosomal location, duplication and synteny analysis

**FIGURE 5:**
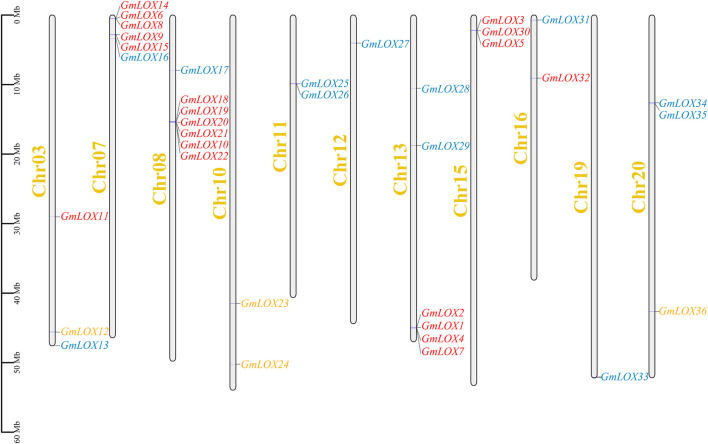
Chromosomal distribution of *GmLOX* family genes in Zhonghuang 13 Note: The scale is chromosome length (Mb). The grey bars represent chromosomes. Chromosome numbers are shown on the left side of the bar. Chr03-20, chromosomes in Zhonghuang 13. The *GmLOX* genes are marked on the right side of the chromosome.

The distribution of genes across chromosomes indicated that the 36 *GmLOX* genes were located on chromosomes 3, 7, 8, 10, 11, 12, 13, 15, 16, 19 and 20, including 3 on Chr3, 7 on Chr7, 7 on Chr8, 2 on Chr11, 1 on Chr12, 6 on Chr13, 3 on Chr15, 2 on Chr16, 1 on Chr19 and 4 on Chr20 (Figure 5). In the figure, 9-*LOX* members are orange font, type Ⅰ 13-*LOX* is red and type II 13-*LOX* is blue. Some of the *GmLOX* genes on chromosomes 7, 8, 11, 13, 15, and 20 are quite close together. Tandem repeats based on positional relationships can be inferred.

**FIGURE 6:**
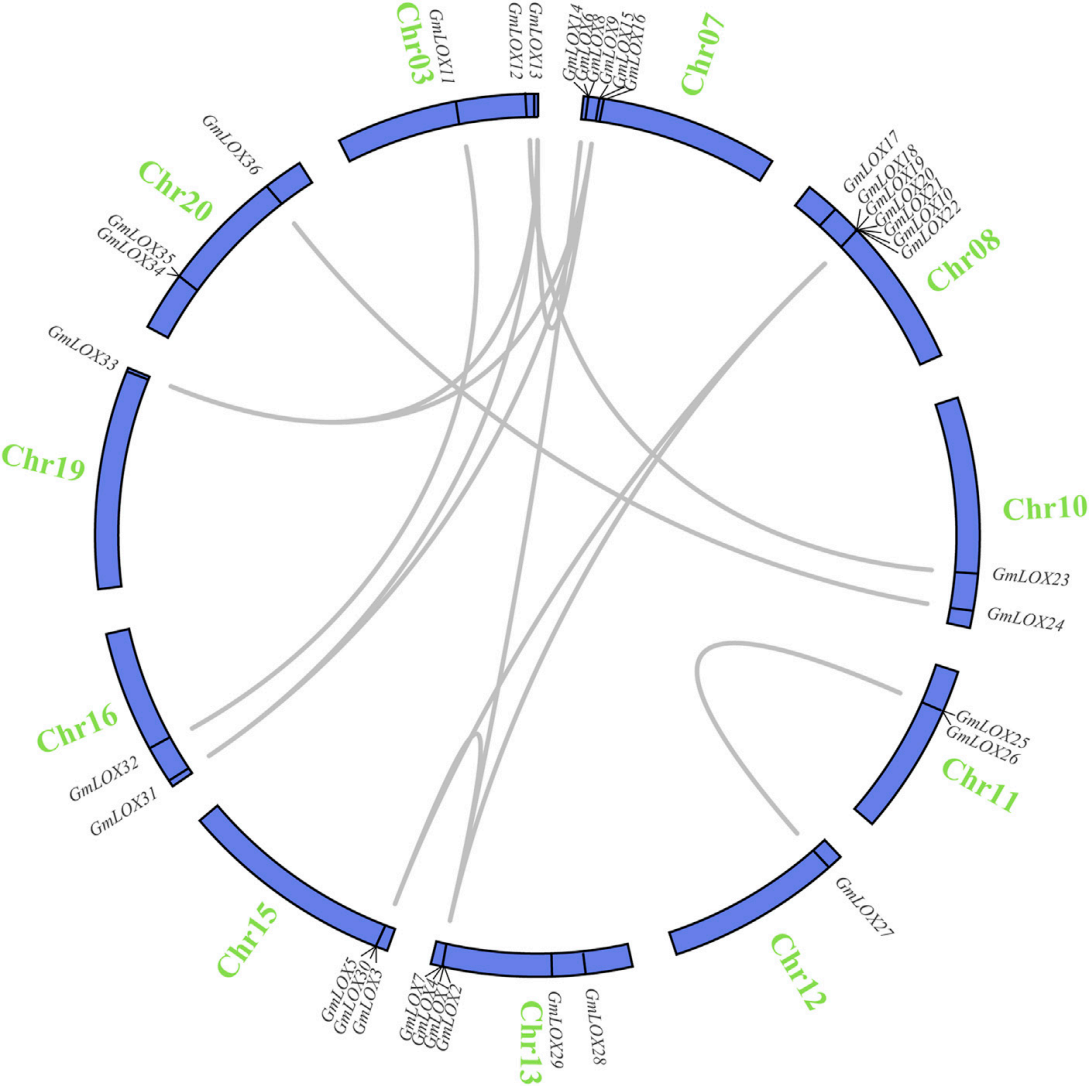
Circle plot of segmental duplicated gene pairs of *GmLOX* genes on chromosomes of Zhonghuang 13 Note: The blue circle bars represent chromosomes. Chromosome numbers are located outside the bar in green. The genes connected by the grey line are deplication gene pairs.

Soybean is an ancient tetraploid plant that underwent two genome-wide duplication events (Song et al., 2017). Approximately 75% of the genes in the soybean genome have multiple copies (Schmutz et al., 2010). Duplication analysis of *GmLOX* genes was performed, and a large number of genes were found to be collinear. There were 14 duplicate gene pairs identified among 36 *GmLOX* (Figure 6). This result suggested that the *GmLOX* gene family underwent extensive duplication during the evolution of soybean. Notably, *GmLOX2* and *GmLOX3* were a pair of duplicated genes. In parallel, we calculated the *K*_*a*_/*K*_*s*_ of these duplicated gene pairs (Table 2). The *GmLOX* gene family was subjected to purifying selection during the evolution of soybean.

**TABLE 2:** Estimation of the K_a_/K_s_ ratio of homologous gene pairs in Zhonghuang 13.

Duplicated gene pairs	K_a_	K_s_	K_a_/K_s_
*GmLOX16&GmLOX31*	0.008914	0.113028	0.078862
*GmLOX24&GmLOX36*	0.024543	0.171402	0.14319
*GmLOX13&GmLOX16*	0.103913	0.835826	0.124324
*GmLOX13&GmLOX31*	0.110218	0.845652	0.130335
*GmLOX16&GmLOX33*	0.106311	0.816789	0.130158
*GmLOX2&GmLOX3*	0.155246	1.16379	0.133397
*GmLOX18&GmLOX3*	0.146551	0.996579	0.147054
*GmLOX12&GmLOX23*	0.132332	0.715231	0.185019
*GmLOX25&GmLOX27*	0.028478	0.134143	0.212292
*GmLOX18&GmLOX2*	0.151264	0.838906	0.180311
*GmLOX10&GmLOX4*	0.172449	0.872106	0.197738
*GmLOX14&GmLOX1*	0.199831	1.1053	0.180794
*GmLOX11&GmLOX32*	0.025843	0.104807	0.246575
*GmLOX13&GmLOX33*	0.027918	0.103846	0.268836

Note: *K*_*a*_, nonsynonymous substitution rates; *K*_*s*_, synonymous substitution rates; *K*_*a*_/*K*_*s*_, the ratio of *K*_*a*_ *versus K*_*s*_ mutation as an indicator to determine the selective pressure or strength on a protein-encoding gene. *K*_*a*_/*K*_*s*_ = 1 shows no selection, *K*_*a*_/*K*_*s*_ < 1 indicates negative or purifying selection and *K*_*a*_/*K*_*s*_ > 1 denotes positive or Darwinian selection.

### Prediction of cis-regulatory elements in the *GmLOX* promoters

**FIGURE 7:**
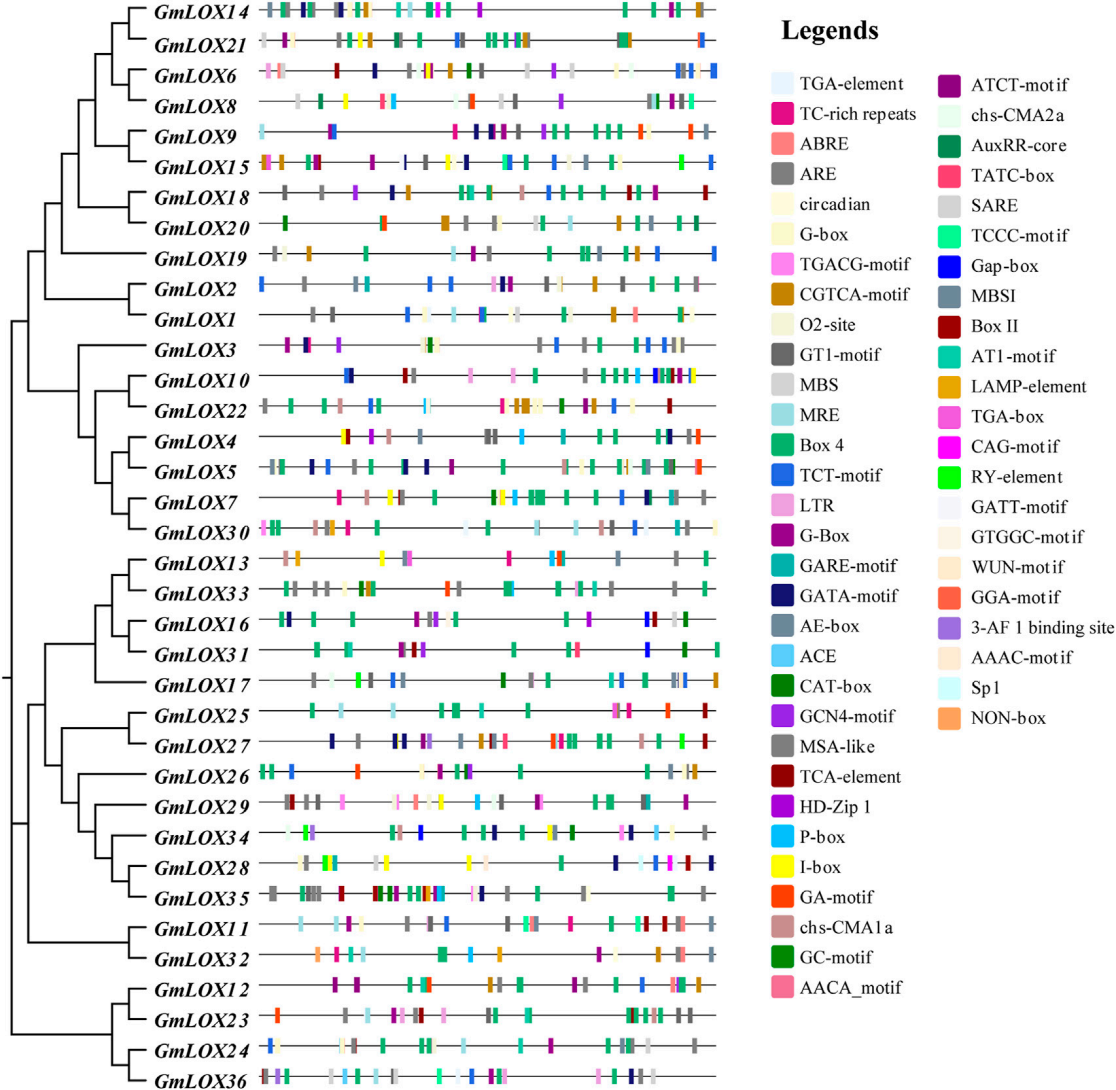
Analysis of cis-elements in the promoters of *GmLOX* genes Note: 2,000 bp upstream from the transcription initiation site was intercepted for analysis, and the sequence lengths in the figure all represent 2000. Response elements involve stress, growth and development, hormones, light response and so on.

To explore the regulatory mechanism of *GmLOX* genes, the cis-regulatory elements of promoter sequences were predicted. As shown in Figure 7, a total of 51 cis-acting elements were identified, including nine growth- and development-related cis-elements, 12 hormone-responsive cis-elements, five stress-responsive cis-elements and 25 light-responsive cis-elements. The hormonal response cis-elements include abscisic acid responsiveness, auxin responsiveness, gibberellin responsiveness, MeJA responsiveness, salicylic acid responsiveness and flavonoid biosynthetic gene regulation. Stress-responsive cis-elements included anaerobic induction, drought inducibility, low-temperature responsiveness and defense and stress responsiveness. A summary of the number of functional cis elements of each gene is given in Supplementary Table S4. These results suggest that the GmLOX gene family may play a role in growth and development and its response to light, hormones, and stress.

### Evolution of *LOX* genes between wild soybean (*G. soja* W05) and Zhonghuang 13

**FIGURE 8:**
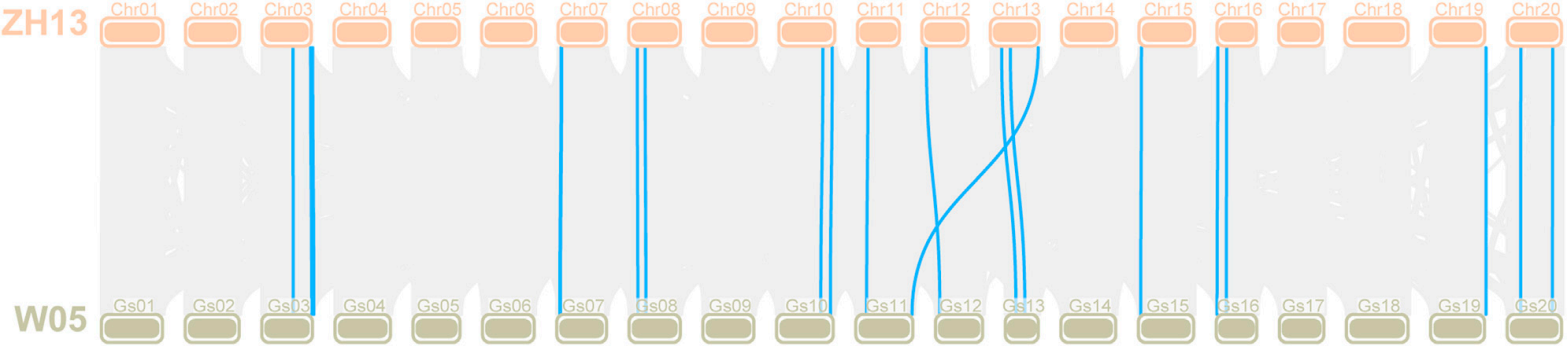
Collinearity analysis of the *LOX* gene between Zhonghuang 13 and *G. soja* W05 Note: The orange bars at the top marked with Chr01-20 represent chromosomes in Zhonghuang 13, and the green bars labelled Gs01-20 at the bottom represent the wild soybean (*G. soja* W05) chromosomes. Orthologous gene pairs of genes in the two varieties are linked by blue highlighted lines.

*G. soja* is the undomesticated ancestor of *G. max,* and soybean domestication is complicated (Kim et al., 2010), indicating that it is of great significance to explore the changes in the *LOX* gene during soybean domestication. The *LOX* gene family members of *G. soja* W05 were identified according to the method described above. Through BLAST alignment with *LOX* genes in Williams 82 and Zhonghuang 13, we found that two tandem genes were annotated into one gene in W05. We manually modified the *LOX* gene annotations in W05 that were identified as faulty. The evolution of *LOX* genes was observed by identifying the orthologous gene pairs of *LOX* genes between *G. soja* W05 and Zhonghuang 13 (Figure 8). The previous results (Xie et al., 2019) of chromosome sequence comparison between Zhonghuang 13 and W05 showed that translocation occurred on chromosomes 11 and 13 of soybean. The translocation segment contains a gene cluster of the *LOX* gene family, which is on chromosome 11 in W05 but on chromosome 13 in Zhonghuang 13. The results of calculating the *K*_*a*_/*K*_s_ and *K*_*n*_/*K*_*s*_ of the orthologous gene pairs are shown in Supplementary Table S5. The *K*_*a*_/*K*_*s*_ values of most orthologous gene pairs were less than 1, while a few gene pairs had very high values. The *K*_*n*_/*K*_*s*_ values of both the intron sequence and promoter sequence were very low, indicating that the noncoding sequences of the *LOX* gene between the two varieties were relatively conserved.

### Expression analysis based on RNA-Seq data in different tissues of soybean

**FIGURE 9:**
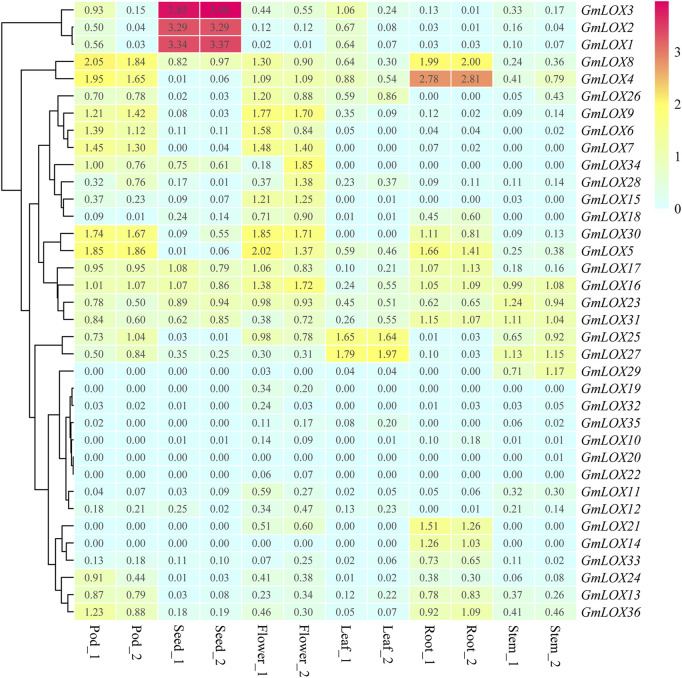
Heatmap of the expression patterns of *GmLOX* genes in different tissues based on transcriptome data Note: Root_1 and Root_2 are two biological duplications in roots (*R* ^2^ > 0.85), and so on for other organizations. The log10 conversion value of expression values +1 (transcripts per million, TPM) was used for plotting. Light blue represents low expression, yellow represents medium expression, and red represents high expression.

The expression pattern of each *GmLOX* is shown in Figure 9, and the genes were arranged based on hierarchical clustering analysis. The heatmap of RNA-Seq analysis reflects that many *GmLOX*s appeared at low transcript abundance levels. The expression levels of *GmLOX1*, *GmLOX2* and *GmLOX3* in seeds were particularly high, and *GmLOX4* in soybean roots was extremely high. The RNA-Seq atlas data revealed that some *GmLOX* genes are expressed specifically in soybean tissues. *GmLOX6*, *GmLOX7* and *GmLOX9* are specifically expressed in flowers and pods, *GmLOX21* and *GmLOX14* are specifically expressed in soybean roots, and *GmLOX29* is specifically expressed in stems. However, two genes (*GmLOX20* and *GmLOX22*) had very low expression levels and could be considered unexpressed in our samples.

### RNA isolation and quantitative real-time PCR (qRT‒PCR)

**FIGURE 10:**
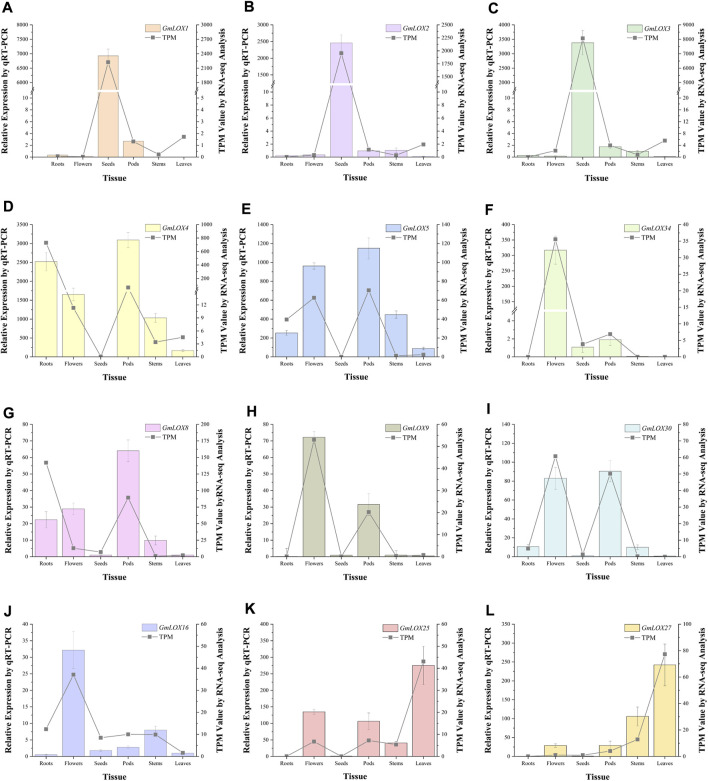
Twelve *GmLOX* genes were selected for qRT‒PCR analysis in different tissues Note: The left vertical axis represents the relative expression obtained by qRT‒PCR analysis, which is shown by the bar graphs in the figure. The standard deviation was calculated from the results of three independent experiments. The right vertical axis is the TPM value calculated by RNA-seq analysis, which is presented in the form of line graphs in the figure. Labels **(A–L)** show the qRT-PCR analysis results of *GmLOX1*, *GmLOX2*, *GmLOX3*, *GmLOX4*, *GmLOX5*, *GmLOX34*, *GmLOX8*, *GmLOX9*, *GmLOX30*, *GMLOX16*, *GmLOX25*, and *GmLOX27* genes, respectively.

By analyzing the qRT‒PCR data of the three reference genes, the expression levels of the *ELF1b* gene were significantly different in each tissue, while the expression levels of the *CPY2* and *ACT* genes tended to be stable, so the geometric mean of the Ct values of these two genes was used to calculate the expression of 12 *GmLOX* genes. Due to the large difference in *GmLOX* gene expression, samples with appropriate expression levels for each gene were selected as calibration samples. The qRT‒PCR results showed that *GmLOX* had extremely significant tissue-specific expression, which was represented by *GmLOX1*, *GmLOX2* and *GmLOX3*, and the expression of these three genes was super hyper-expressed in seeds (Figures 10A–C). The expression levels of *GmLOX4* and *GmLOX5* were low in seeds but high in other tissues to varying degrees (Figures 10D,E). The expression of *GmLOX34* was only high in flowers (Figure 10F). The expression levels of *GmLOX8*, *GmLOX9* and *GmLOX30* were fairly low in seeds and leaves; *GmLOX8* and *GmLOX30* were expressed to different degrees in other tissues, while *GmLOX9* had a strong specific expression in flowers and pods (Figures 10G–I). The expression patterns of *GmLOX25* and *GmLOX27* were also similar in that they were not expressed in roots and seeds (Figures 10J,K). The expression of *GmLOX16* is high in flowers and is also found in other tissues (Figure 10L). The trend of the qRT‒PCR results was consistent with that of the transcriptome analysis.

## Discussion

In soybean, lipoxygenase protein is encoded by a multigene family (Liu Y et al., 2020). In this study, we identified lipoxygenase gene family members in Zhonghuang 13. Although Zhonghuang 13 is the most complete soybean genome at present, its gene structure annotation results are not very accurate for practical use. BUSCO scores assessed the genome assembly up to 99.7%, but the annotated protein-coding genes were only up to 96.3%. Therefore, we first calibrate the annotation of *LOX* genes manually. In the phylogenetic analysis, to confirm the accuracy of the *LOX* gene family members results in alien species, we verified the existing but unidentified results in the literature (Umate, 2011; Chen et al., 2015) and deleted the genes lacking the PLAT domain or lipoxygenase domain. The results confirmed that the numbers of *LOX* genes in *O. sativa* and *P. trichocarpa* were 11 and 18, respectively. Consistent with previous research (Liu et al., 2011; Shaban et al., 2018; Wang et al., 2019), the *GmLOX* genes were divided into three groups (type Ⅰ 13-LOX, type II 13-LOX, and 9-LOX) according to protein structure and similarity. The proteins of these subfamilies bind to substrates at different locations. The carbon atom nine of the hydrocarbon main chain of 9-LOX fatty acids binds to substrates and mainly participates in the fat oxidation reaction of linoleic acids (LA, 18:2). Carbon atom 13 of the hydrocarbon main chain of 13-LOX fatty acids binds to substrates and is mainly involved in the fat oxidation reaction of linolenic acids (LeA, 18:3) (Podolyan et al., 2010). Combined with the analysis of chromosome location, more than half of the genes are concentrated on chromosomes 7, 8, and 13 and exist as gene clusters. Almost all the genes of the gene clusters belong to the 13-LOX subfamily, indicating that specific amplification of the soybean *LOX* gene family occurred in the evolution of species. Tandem duplication occurs for a number of reasons. It is currently believed that tandem duplication is presumed to arise through unequal chromosomal crossing over (Leister, 2004), which is responsible for much of the tandem duplication gene number and allelic variation within species. Tandem duplicated genes are likely to be important for adaptive evolution to rapidly changing environments (HUANG et al., 2022). Some studies have shown that the formation of tandem duplication genes during evolution is related to immunity and stress (Rodgers-Melnick et al., 2012), which verifies the viewpoint that the *LOX* genes is related to stress (Rosahl, 1996). Relevant studies have found transposable element sequences in *LOX* genes of wheat (Garbus et al., 2009). In this study, the RepeaMasker program (Tempel, 2012) was used to analyze the repeat sequence in the Zhonghuang 13 genome and found that LTR (long-term relationship) sequences existed in more than 70% of the *GmLOX* genes, suggesting that transposition may be one of the reasons for the amplification of the *GmLOX* gene. In addition, pseudogenes may also be formed due to the loss of normal function caused by improper transposon insertion sites or sequence mutation. The spatiotemporal expression data of genes also reflect the hypothesis that many genes are not involved in transcriptional expression. In the comparison of *LOX* genes between Zhonghuang 13 and Williams 82, two *LOX* genes that have been eliminated by mutations supported the above view. Gene duplication plays an important role in the generation of new genes and sub-functionalization or new functionalization (Zhang et al., 2016a). There are a large number of duplicated gene pairs in the *GmLOX* gene family, and the duplicated genes have undergone purification selection pressure. In the conserved motifs, motif E is a specific sequence of the LOX family, which contains a sequence of 38 amino acid residues, His-(X)4-His-(X)4-His-(X)17-His-(X)8-His (Supplementary Figure S3). All GmLOX proteins contain this highly conserved structure (Zhang Z et al., 2006). In the prediction of GmLOX subcellular localization, these genes were mainly distributed in the cytoplasm and chloroplast. This result was consistent with that of subfamily clustering. Most of the Type II 13-LOX genes that had a chloroplast targeting peptide were located in chloroplasts, which may be related to the structure of the gene sequence. Relevant reviews indicate that Type II-LOX proteins are chloroplast proteins that carry N-terminal plastid transport peptides, while Type I-LOX without transport peptides usually have cytoplasmic localization (Zhang et al., 2014). It could be inferred that different lipoxygenases play a catalytic role in different positions.

In *A. thaliana*, *AtLOX* genes belong to only the 9-LOX and type II 13-LOX subfamilies (Bannenberg et al., 2009). In the 9-LOX subfamily, *GmLOX12* and *GmLOX23* are clustered with *AtLOX1*, and *GmLOX24* and *GmLOX36* are associated with *AtLOX5*. It has been reported that the products of 9-LOX genes in *A. thaliana* can protect plants from various biological and abiotic stresses (Vellosillo et al., 2007). The synthesis of JA compounds requires participation of the *LOX* gene (Ruan et al., 2019). A relevant article shows that *AtLOX1* plays an important regulatory role in the synthesis of metabolites such as JA in response to pathogen attack (Chen et al., 2015), which implies that *GmLOX12* and *GmLOX23* may participate in the formation of JA. The response cis-regulatory elements associated with the regulation of methyl jasmonate (MeJA) were identified in the analysis of the *GmLOX* gene promoter region. Nevertheless, further research and confirmation are needed for the specific potential mechanism of this function. Some studies indicated that external application of MeJA had a certain effect on *LOX* gene expression. In a study of the *LOX* gene family in *C. lanatus*, the most significant increases were observed for *ClLOX7* upon JA treatment, suggesting that *ClLOX7* plays a primary role in JA signaling in leaves and roots (Liu J et al., 2020). Exogenous MeJA treatment was found to significantly enhance the transcript expression of most *SpLOX* genes belonging to the 13-LOX subfamily (Upadhyay et al., 2020).

Wild soybean is a close relative ancestor of cultivated soybean, and they both belong to Leguminosae, *Papilionoideae*, *Glycine*, and *Soja* (Zhang et al., 2016b). Cultivated soybean is the result of artificial selection according to people’s needs. In contrast, there are more diverse original genes in wild soybean. At present, many studies trace the evolutionary origin of genes by comparing the differences between wild soybean and cultivated soybean (Rehman et al., 2021). The *LOX* gene of wild soybean (*G. soja* W05) was also identified and compared. Notably, the translocation segment between chromosome 11 and chromosome 13 contained a *LOX* gene cluster including four *LOX* genes. Many studies have shown that noncoding sequences play an important regulatory role in biological evolution (Zhang, 2022). The *K*_*n*_ */K*_*s*_ values of intron and promoter sequences are very low, indicating that the noncoding sequence of the *GmLOX* gene is highly conserved. For the coding sequence, the Ka/Ks results showed that most gene pairs were subject to purification selection pressure. However, there were several gene pairs for which *K*_*a*_ */K*_*s*_ values were far greater than 1. We speculated that on the one hand, these genes may have experienced positive selection, or on the other hand, they may have been caused by population polymorphism. Additionally, during the evolution of species, there would be regional population differences. The species that evolved from the same region would have the highest similarity with the ancestors of the region, but there would be more SNP (single nucleotide polymorphism) loci with the ancestors of other regions. It could be speculated that these regions may come from different populations that have wide nucleic acid polymorphisms, resulting in *K*_*a*_/*K*_*s*_ values of individual genes far greater than 1. We analyzed the location of introns and exons of *LOX* genes in W05. The result is very similar to that of Zhonghuang 13. Theoretically, in the recent evolution of eukaryotes, a high intron loss rate is a more common phenomenon (Roy and Penny, 2007). The number of introns of *LOX* genes in W05 and Zhonghuang 13 basically did not change. *LOX* genes are very conserved. In the long-term domestication and improvement of soybeans, only a small number of genetic resources were selected (Huihui et al., 2018). We speculate that the *LOX* gene family may not belong to strong artificial selection loci.

The cis-regulatory elements in the *GmLOX* promoters mainly include defense and stress, circadian control, development and tissue specificity, light and hormones. The number of light response elements was the largest, and relevant studies have investigated the relationship between the expression of the *LOX* gene and light. In the study of the expression pattern of the *LOX* gene in response to light in *Fagopyrum tataricum*, red and blue light induced the expression of *FtLOX1* and *FtLOX7*, whereas *FtLOX4* and *FtLOX6* were inhibited by light. *FtLOX5-7* expression levels were the highest under far-red light (Ji et al., 2020). Previous studies have also shown that *LOX* expression is strongly associated with the application of plant hormones such as MeJA, salicylic acid, abscisic acid, and ethylene. A study of the differential expression of the *LOX* gene family in ripening kiwifruit found that the expression levels of *AdLOX2*, *AdLOX3* and *AdLOX4* were negatively correlated with ethylene accumulation, and ethylene application promoted a decrease in the expression levels (Zhang B et al., 2006). In *Taxus chinensis*, *TcLOX1* was upregulated by MeJA and abscisic acid, while *TcLOX2* was upregulated by ABA (Li et al., 2012). The verification of the above functions of *GmLOX* genes needs to be further studied.

In qRT‒PCR analysis, some reference genes or housekeeping genes may sometimes respond considerably to changes in experimental conditions or tissue types (Gutierrez et al., 2008; Hu et al., 2009). Therefore, multiple internal reference genes were selected to reduce the influence of uncertainty. In our study, three reference genes were selected: *GmACT*, *GmCYP2*, and *GmELF1B*. The *ACT* gene is involved in the formation of cytoskeletal structural proteins. The *CYP2* gene is involved in protein folding, and the *ELF1B* gene is involved in translation elongation. Previous studies have shown that unique expression patterns occur in some *LOX* genes, which can provide a favorable starting point for studying the mechanism and physiological function of *LOX* genes. For example, analysis of *Oep2LOX1* expression levels in different olive tissues from Picual and Arbequina cultivars showed that *Oep2LOX1* expression was higher in leaves but lower in other tissues (Padilla et al., 2012). These genes exist in different tissue sites, which may indicate differences in function. Transcriptional data analysis of different soybean tissues showed that *GmLOX* gene expression had significant tissue specificity. Represented by *GmLOX1*, *GmLOX2* and *GmLOX3*, the expression levels in seeds were particularly high. Wang et al. (2020) obtained mutants without *GmLOX1*, *GmLOX2* and *GmLOX3* using a pooled CRISPR‒Cas9 system, confirming the role of these three LOXs in causing the beany flavor. Related articles have shown that LOXs stored in seeds can be used as nutritional storage proteins and participate in the process of seed maturation and seedling growth (Viswanath et al., 2020). It can be speculated that these three *GmLOX* genes with high expression in seeds may play a role in seed maturation and seedling growth. These findings provide important clues for further exploring the function of *GmLOX* genes in the future.

## Conclusion

In this study, 36 *GmLOX* genes were identified in Zhonghuang 13. We modified the annotation information of four *GmLOX* genes in Zhonghuang 13 by combining transcriptome analysis data with *LOX* gene information from William 82. We verified the existence of *GmLOX1* at both the gene and protein levels. Phylogenetic analysis showed that the *LOX* gene was mainly divided into three clusters: type I 13-LOX, type II 13-LOX, and 9-LOX. The type I 13-LOX gene of soybean accounted for a whole branch. Combined with gene localization and collinearity, it could be speculated that the type I 13-LOX subfamily of *GmLOX* genes may have replicated on a large scale. The *GmLOX* genes were subjected to negative selection pressure during evolution. The highly conserved characteristics of *GmLOX* genes were confirmed by both gene structure and conserved motifs. There are many cis-regulatory elements, including those related to stress response, growth and development, hormone response and light response, in the promoter region. The *K*_*a*_ */K*_*s*_ analysis of coding sequences and *K*_*n*_ */K*_*s*_ analysis of noncoding sequences (intron sequence and promoter sequence) of orthologous gene pairs between Zhonghuang 13 and *G. soja* W05 showed that the noncoding sequences of *GmLOX* genes were highly conserved, and the coding sequences of most *GmLOX* genes were subject to negative selection. The spatial expression of *GmLOX* genes was studied and the results showed that some genes had obvious tissue-specific expression. These findings lay a foundation for further exploration of the function and evolution of the *LOX* gene.

## Data Availability

The datasets presented in this study can be found in online repositories. The names of the repository/repositories and accession number(s) can be found in the article/Supplementary Material. RNA-Seq data were submitted to the NCBI Sequence Read Archive (SRA) under accession numbers SRR21075390 to SRR21075406 (17 accession numbers in total).

## Supplementary material

Supplementary Table S1 Primers for 12 *GmLOX* genes and three internal reference genes.

Supplementary Table S2 Homologous gene pairs between Zhonghuang 13 and Williams 82.

Supplementary Table S3 Gene names corresponding to sequence IDs in four outgroup species.

Supplementary Table S4 The summary of functional cis elements number of each *GmLOX* gene.

Supplementary Table S5 Estimation of *K_a_/K_s_* and *K_n_/K_s_* of orthologous gene pairs between *G. soja* W05 and Zhonghuang13.

Supplementary Data S1
*GmLOX* genes coding sequences.

Supplementary Data S2
*GmLOXs* protein sequences.

Supplementary Data S3
*GmLOX* genes promoter sequences -2,000 bp upstream of the *GmLOX* translation initiation codon.

Supplementary Data S4 Raw sequencing results of *GmLOX1* gene full-length sequence.

Supplementary Figure S1 Two *LOX* genes terminated prematurely of protein translation due to mutation.

Supplementary Figure S2 Sequence details of 10 motifs.

Supplementary Figure S3 Conserved structure constituted by 38 amino acid residues in *LOX* gene.
