# Supplementary figures and images for: Genome-wide identification and analysis of LOX genes in soybean cultivar “Zhonghuang 13”
Source: Front Genet. 2022 Oct 7;13:1020554. doi: 10.3389/fgene.2022.1020554 (PMC9585170; doi:10.3389/fgene.2022.1020554)

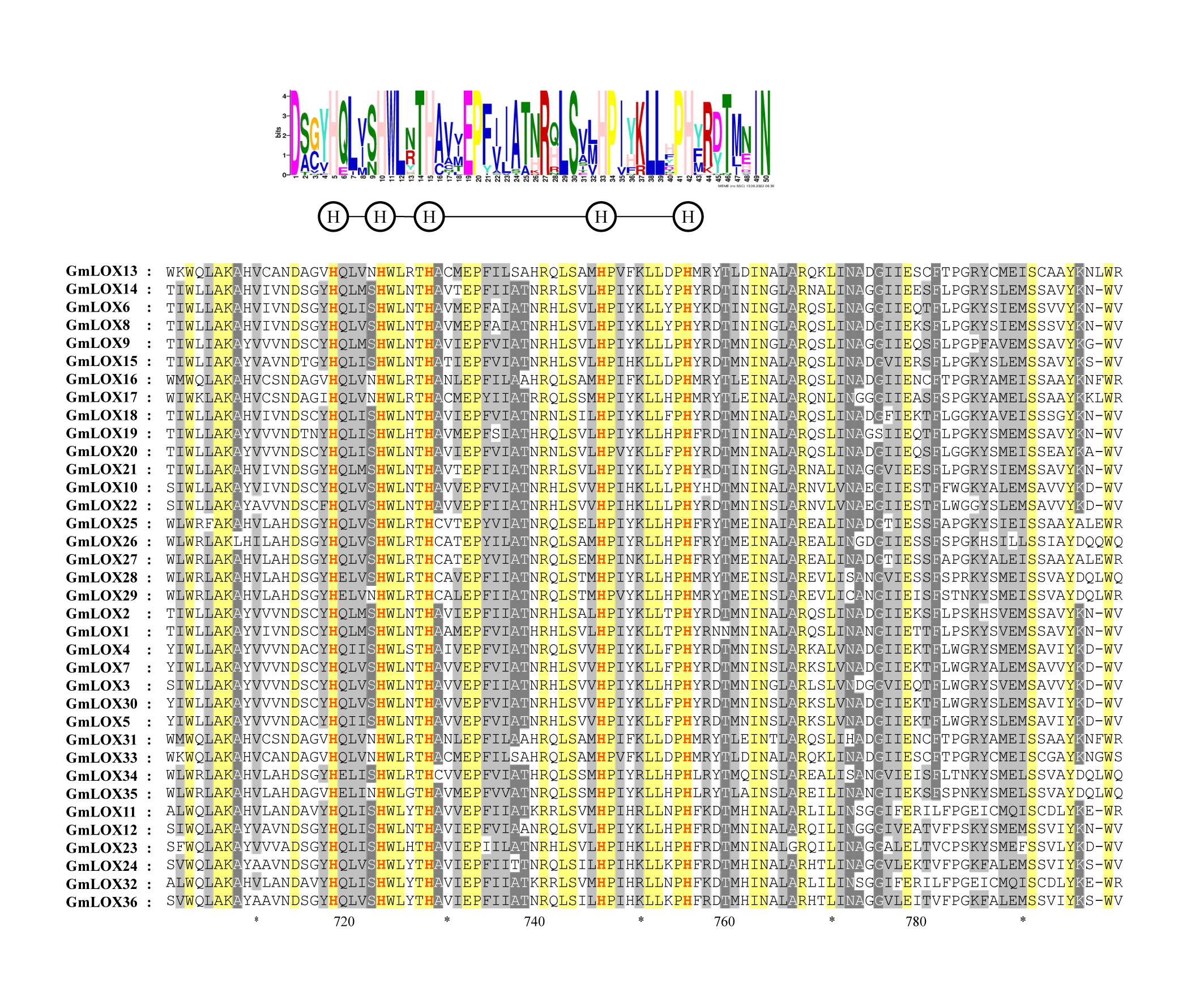

Supplement: Supplementary file 4 [file Image3.TIF]

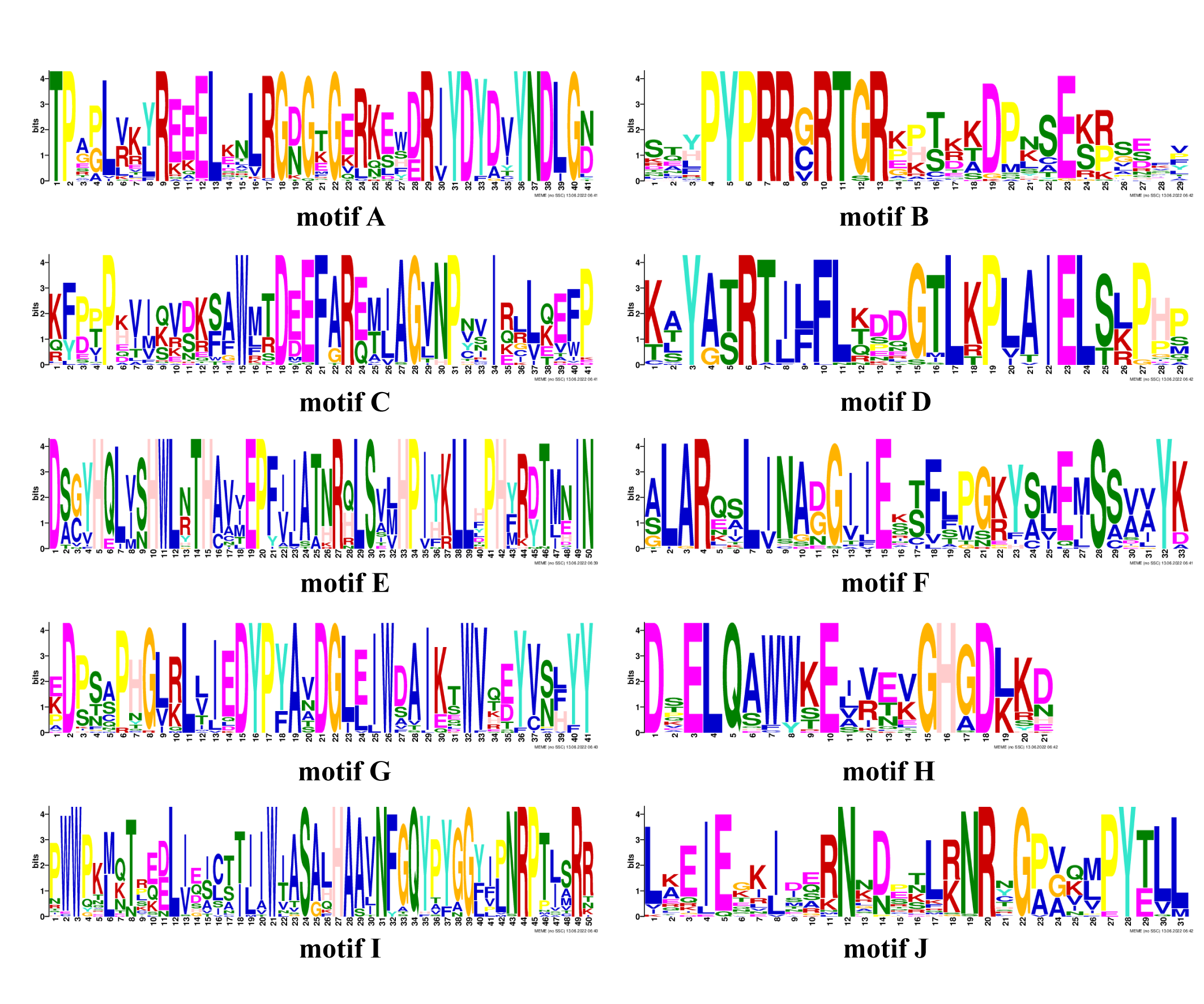

Supplement: Supplementary file 7 [file Image2.TIF]

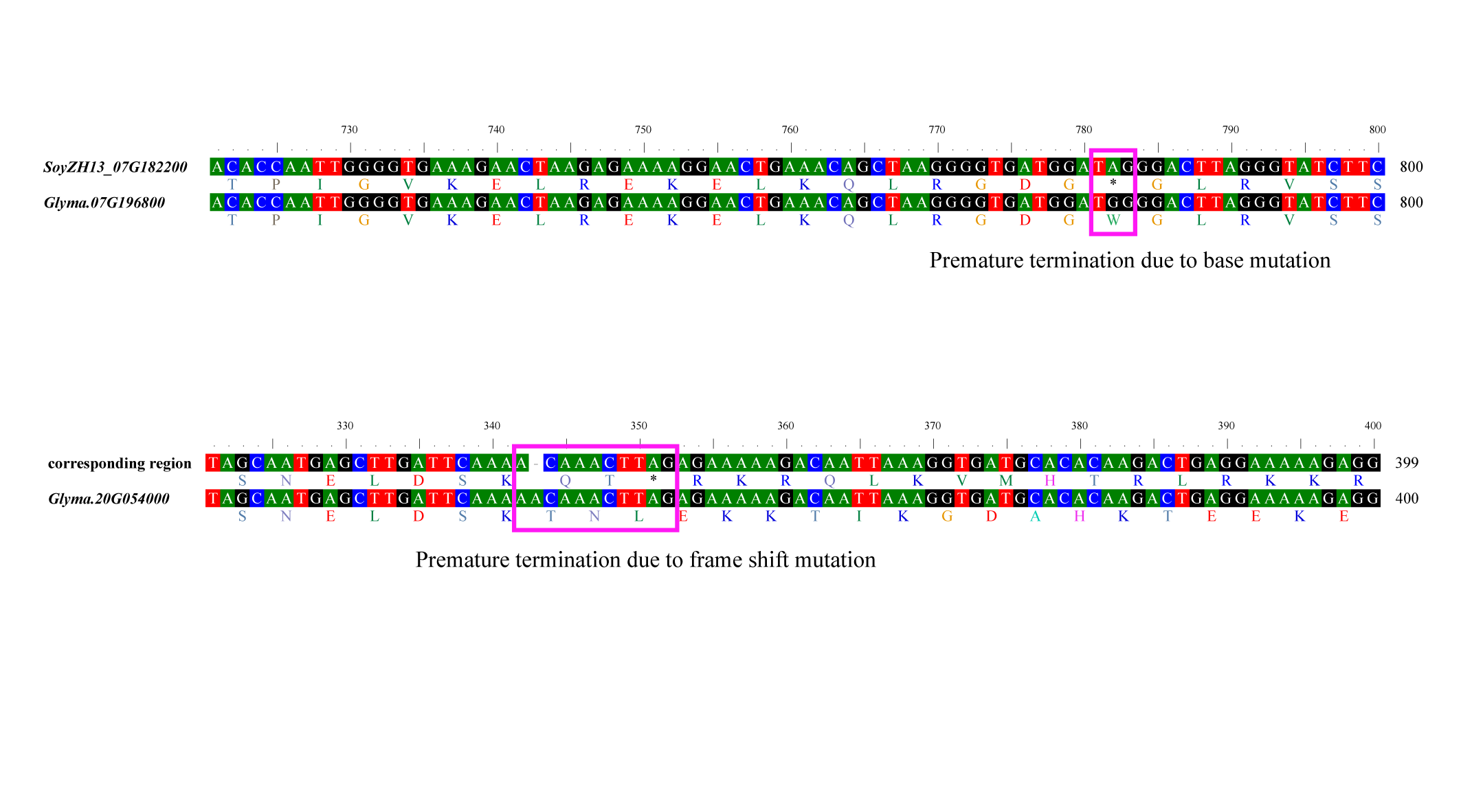

Supplement: Supplementary file 8 [file Image1.TIF]
